# Supplementary figures and images for: Stochastic variation in the FOXM1 transcription program mediates replication stress tolerance
Source: Mol Oncol. 2025 Feb 26;19(6):1633–50. doi: 10.1002/1878-0261.13819 (PMC12161472; doi:10.1002/1878-0261.13819)

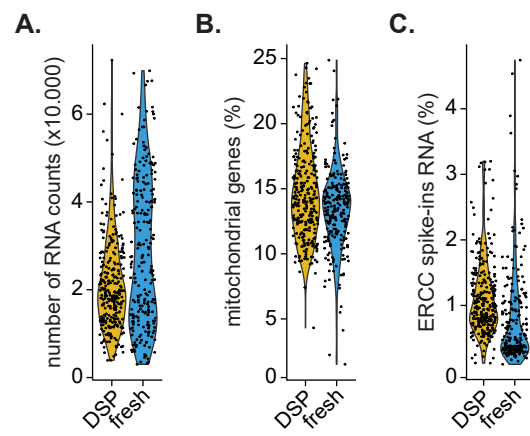

Supplemental Figure 1, related to figure 2

Supplement: Supplementary file 1 — Fig. S1. Related to Fig. 2. (A) Violin plot showing the number of unique RNA (UMI) counts per cell in DSP‐fixed and fresh cells RPE‐HRASG12V cells. (B) Violin plot showing the percentage of RNA counts mapping to mitochondrial genes as percentage of total counts detected in DSP‐fixed and fresh cells RPE‐HRASG12V cells. C Violin plot showing ERCC spike‐in RNA counts as percentage of total counts detected in DSP‐fixed and fresh RPE‐HRASG12V cells. [file MOL2-19-1633-s005.pdf]

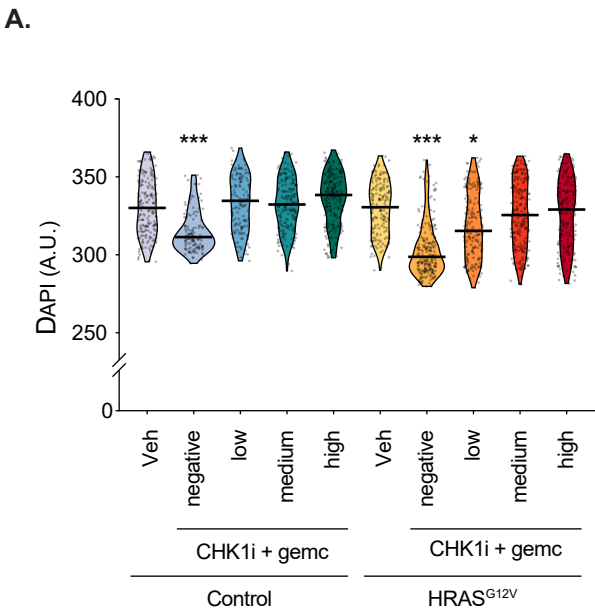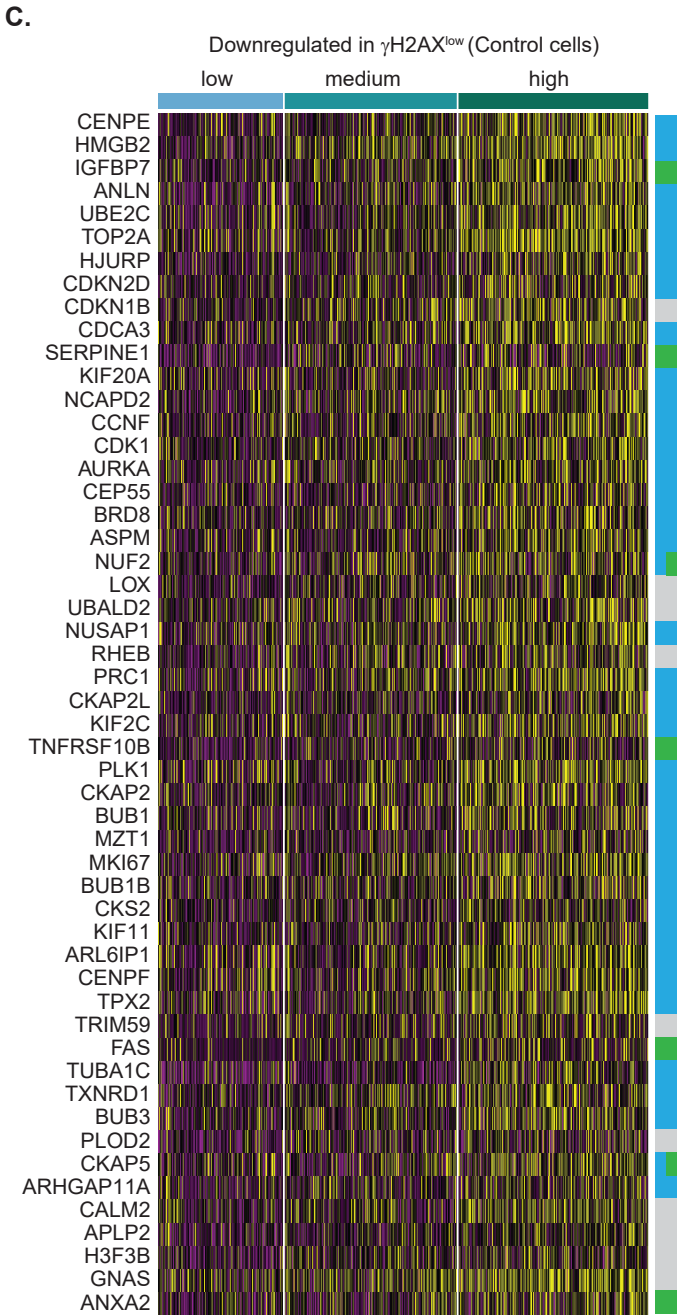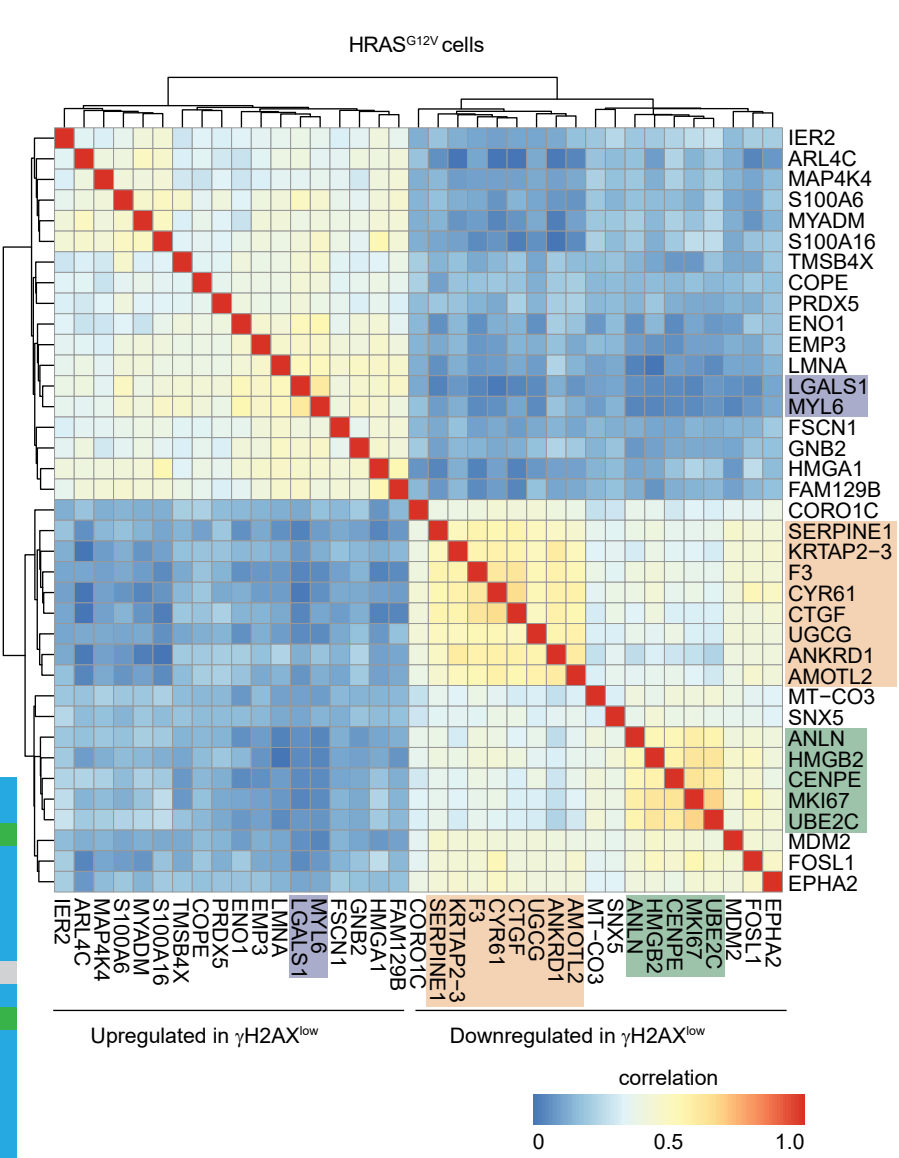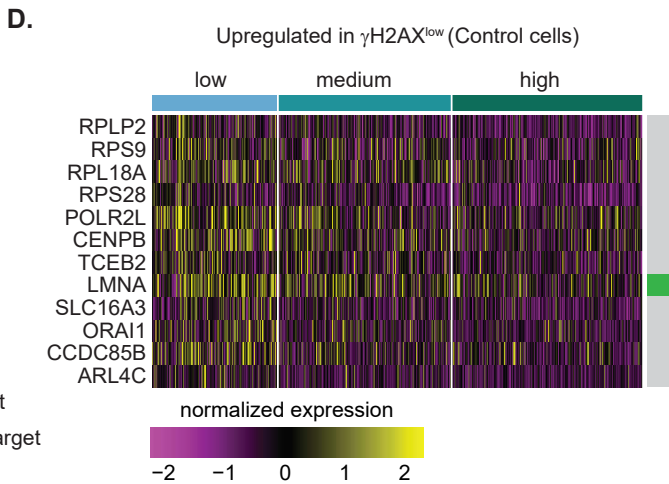

Supplemental Figure 2, related to figure 4

Supplement: Supplementary file 2 — Fig. S2. Related to Fig. 4. (A) The DAPI fluorescence intensity in individual FACS‐sorted cells shown in a violin plot. Horizontal lines represent median DAPI levels in each group. Wilcoxon rank sum tests for multiple group comparisons were performed on control cells and HRASG12V cells separately. ***P < 2E‐16 γH2AX‐negative versus all other groups, *P < 0.005 γH2AXlow versus γH2AXmedium and γH2AXhigh. (B) Correlation matrix displaying the correlations in normalized transcript counts between all the differentially expressed genes in RPE HRASG12V γH2AXhigh versus γH2AXlow cells. Highlighted genes show a correlation coefficient greater than 0.4 with at least 1 other gene. (C) Heatmap of genes differentially expressed and downregulated in γH2AXlow versus γH2AXhigh control RPE cells after treatment with 10 nM CHK1i + 4 nM gemcitabine. The heatmaps represent normalized transcript counts in single‐cell RNA‐sequencing analysis. (D) Heatmap of genes differentially expressed and upregulated in γH2AXlow versus γH2AXhigh control RPE cells after treatment with 10 nM CHK1i + 4 nM gemcitabine. The heatmaps represent normalized transcript counts in single‐cell RNA‐sequencing analysis. Plots in C and D represent n = 141, 194, and 214 cells in the γH2AXlow, γH2AXmedium, and γH2AXhigh groups, respectively. [file MOL2-19-1633-s006.pdf]

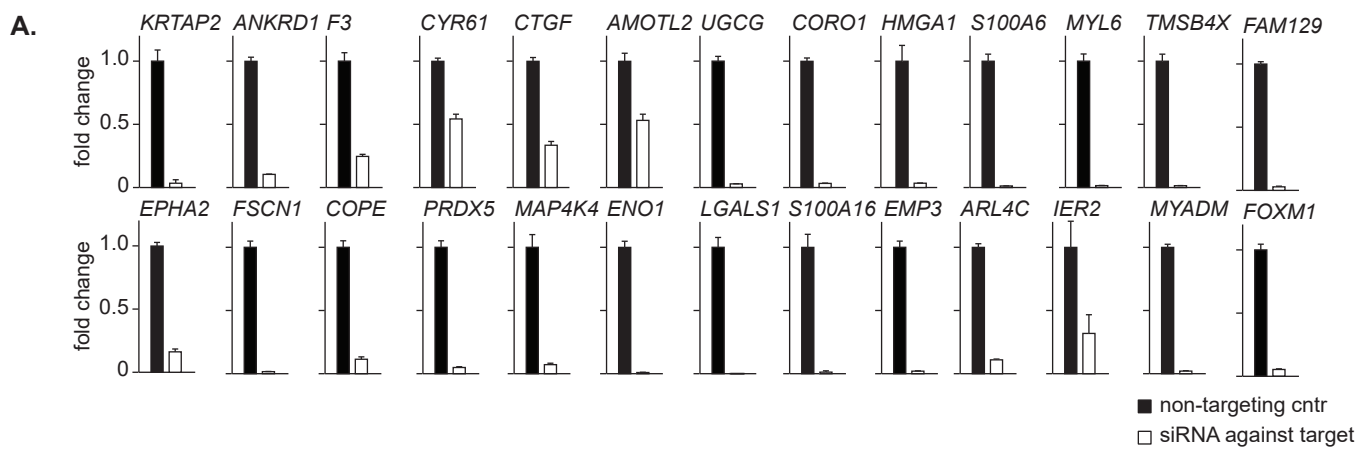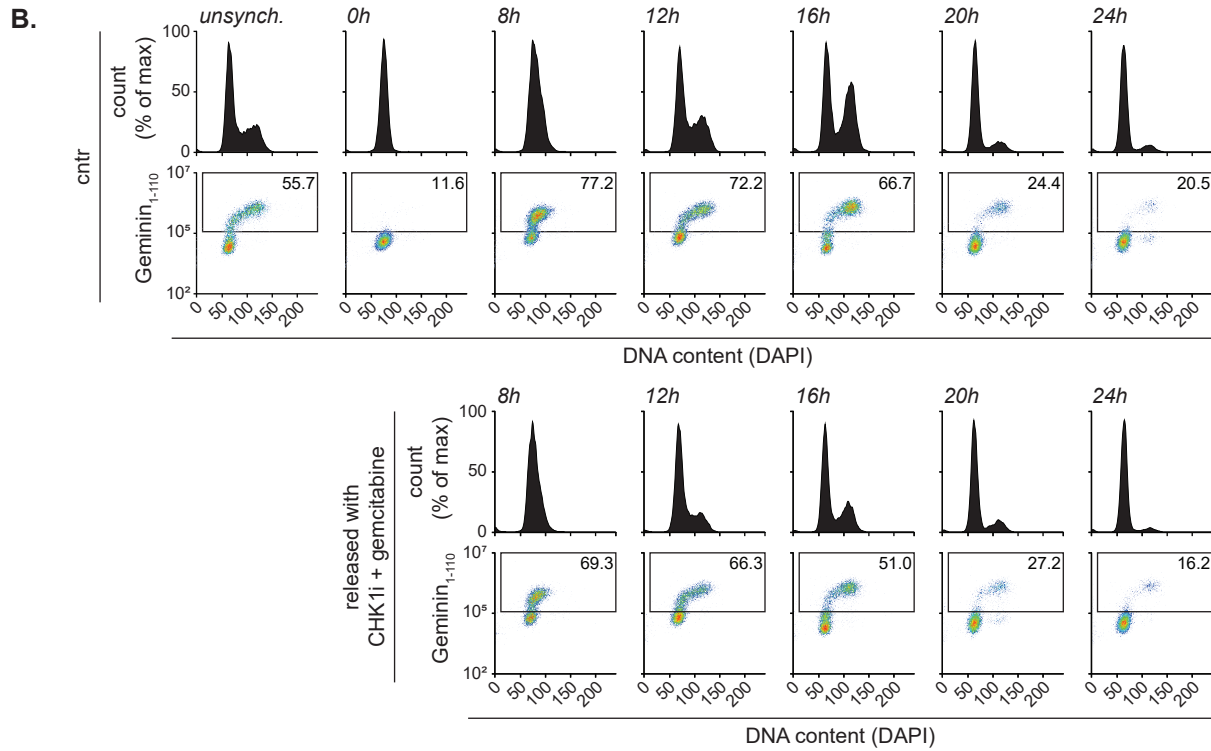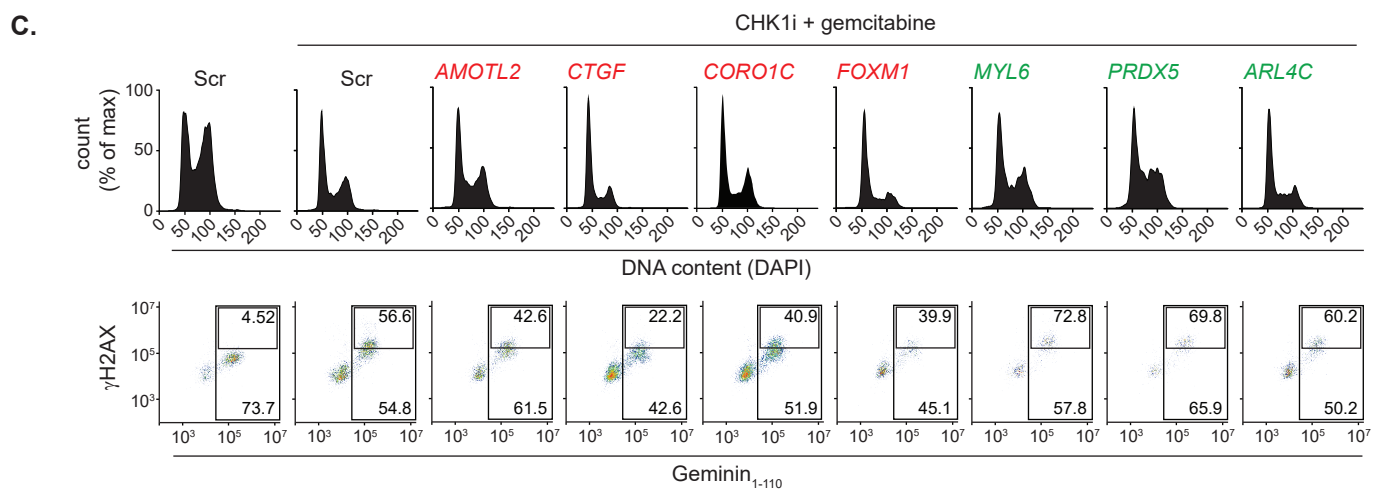

Supplement: Supplementary file 3 — Fig. S3. Related to Fig. 5A. (A) Quantitative PCR of the expression of potential RS‐tolerance conferring genes in RPE‐HRASG12V cells treated with scrambled siRNA or siRNA targeting the gene of interest. Gene expression was normalized to the average of two housekeeping genes (GAPDH, 18S). Bar represents mean ± s.e.m. (B) Flow cytometry data of RPE‐HRASG12V cells unsynchronized, arrested in G1‐phase after 24 h treatment with a CDK4/6i and at indicated hours after release in the presence and absence of CHK1i + gemcitabine to enrich for S/G2‐phase cells. DAPI staining was used to determine cell cycle progression (top row). The relationship between Geminin1‐110 and DAPI is shown in the bottom row. Representative of 2 independent experiments. (C) Flow cytometry data of RPE‐HRASG12V cells with the indicated genes depleted by Smartpools of four individual siRNAs. DAPI staining was used to determine cell cycle progression (top row) and γH2AX staining was used to determine the degree of replication stress (bottom row). Number in bottom right corner of bottom row plots indicates the Geminin1‐110 + cells as percentage of the total cells. Number in the top right corner of bottom row plots indicates γH2AX+ cells as a percentage of Geminin1‐110 + cells. Representative of 2 independent experiments. [file MOL2-19-1633-s002.pdf]

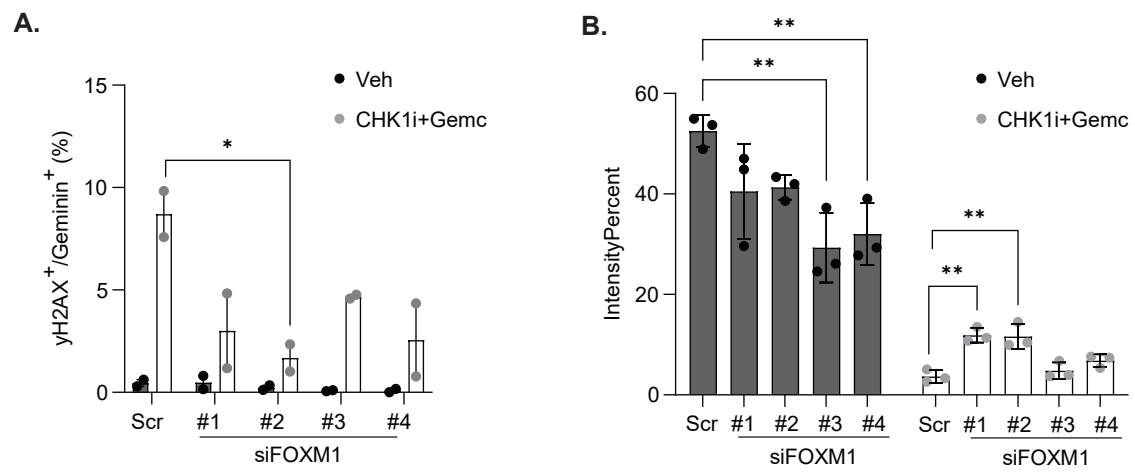

Supplement: Supplementary file 4 — Fig. S4. Related to Fig. 5C and E. (A) Quantification of percent of γH2AX‐positive cells in geminin‐positive cells (representative of cells in S/G2 phase) from two individual experiments. Error bars indicate mean +/− SEM. *P < 0.01. (B) Raw IntensityPercent values calculated using the ImageJ ColonyArea plug‐in. Error bars indicate mean +/− SEM. Significant differences were determined by ordinary One‐way ANOVA followed by Dunnett's multiple comparison test. *P < 0.05, **P < 0.01, N = 3. [file MOL2-19-1633-s001.pdf]

A.

CHK1i + Gemc

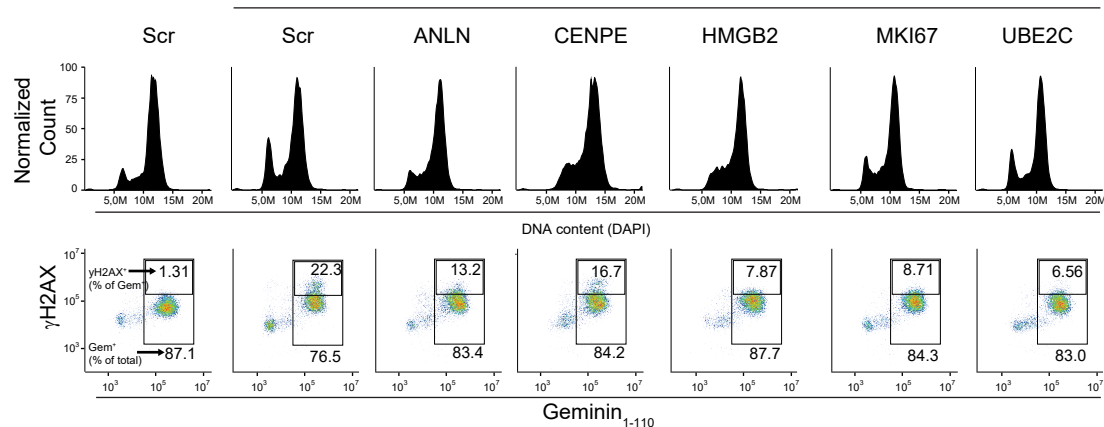

B.

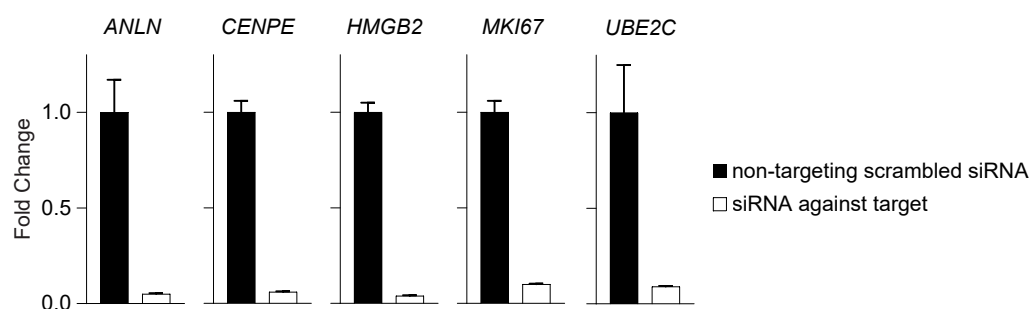

C.

CHK1i + Gemc

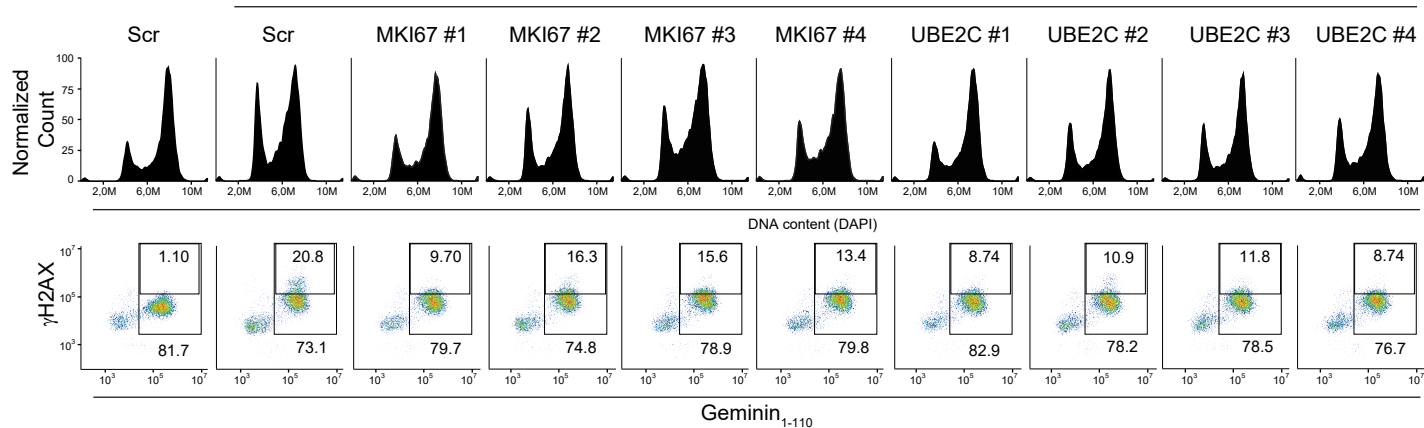

D.

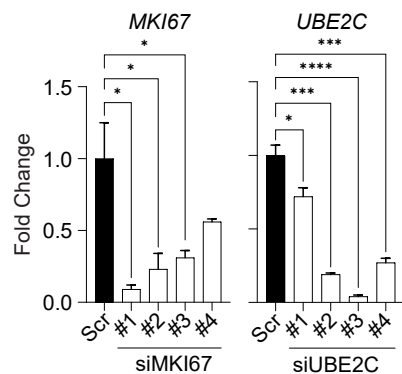

Supplement: Supplementary file 5 — Fig. S5. Related to Fig. 5. (A) DNA damage, measured by γH2AX flow cytometry in S and G2 RPE‐HRASG12V cells treated with indicated FOXM1 target genes depleted by Smartpools of four individual siRNAs. DAPI staining was used to determine cell cycle progression (top row) and γH2AX staining was used to determine the degree of replication stress (bottom row). Number in bottom right corner of bottom row plots indicates the Geminin1‐110 + cells as percentage of the total cells. Number in the top right corner of bottom row plots indicates γH2AX+ cells as a percentage of Geminin1‐110 + cells. (B) Quantitative PCR of the expression of FOXM1 target genes in RPE‐HRASG12V cells treated with scrambled siRNA or siRNA Smartpools targeting the gene of interest. Gene expression was normalized to the average of 2 reference genes (GAPDH, 18S). Bars represent mean ± s.e.m. (C) DNA damage, measured by γH2AX flow cytometry in G2 RPE‐HRASG12V cells treated with four individual siRNAs targeting the FOXM1 target genes MKI67 and UBE2C. DAPI staining was used to determine cell cycle progression (top row) and γH2AX staining was used to determine the degree of replication stress (bottom row). Number in bottom right corner of bottom row plots indicates the Geminin1‐110 + cells as percentage of the total cells. Number in the top right corner of bottom row plots indicates γH2AX+ cells as a percentage of Geminin1‐110 + cells. (D) Quantitative PCR of the expression of the FOXM1 target genes MKI67 and UBE2C in RPE‐HRASG12V cells treated with scrambled siRNA or 4 different individual siRNAs targeting the gene of interest. Gene expression was normalized to the average of 2 reference genes (GAPDH, 18S). Bars represent mean ± s.e.m. [file MOL2-19-1633-s004.pdf]
