# Supplementary material for: Stochastic variation in the FOXM1 transcription program mediates replication stress tolerance
Source: Mol Oncol. 2025 Feb 26;19(6):1633–50. doi: 10.1002/1878-0261.13819 (PMC12161472; doi:10.1002/1878-0261.13819)
Supplement: Supplementary file 6 — Table S1. Differentially expressed genes in γH2AXlow versus γH2AXhigh cells with HRASG12V overexpression. Table S2. Differentially expressed genes in γH2AXlow versus γH2AXhigh control cells. Table S3. Enrichr results of downregulated genes in γH2AXlow HRASG12V cells. Table S4. Enrichr results of downregulated genes in γH2AXlow Control cells. Table S5. Key resources. Table S6. Antibodies for immunoblots and immunofluorescence staining. Table S7. qPCR primers. [file MOL2-19-1633-s003.docx]

**Supplementary information**

**Stochastic variation in the FOXM1 transcription program mediates replication stress tolerance.**

Hendrika A. Segeren, Kathryn A. Wierenga, Elsbeth A. van Liere, Frank M. Riemers, Bart Westendorp

1. **Supplementary Tables**

**Table S1. Differentially expressed genes in γH2AX^low^ versus γH2AX^high^ cells with HRAS^G12V^ overexpression.**

| **Gene** | **avg_log2 FC** | **p_val** | **p_val_adj** | **Upregulated in gH2AX^low^** |
| --- | --- | --- | --- | --- |
| *MYADM* | 0.479 | 3.11E-07 | 4.62E-03 |  |
| *IER2* | 0.460 | 1.72E-06 | 2.55E-02 |  |
| *ARL4C* | 0.448 | 1.35E-07 | 2.00E-03 |  |
| *MAP4K4* | 0.394 | 3.43E-07 | 5.10E-03 |  |
| *PRDX5* | 0.376 | 2.08E-06 | 3.10E-02 |  |
| *GNB2* | 0.365 | 3.60E-07 | 5.35E-03 |  |
| *COPE* | 0.354 | 3.69E-07 | 5.48E-03 |  |
| *FSCN1* | 0.343 | 4.05E-07 | 6.02E-03 |  |
| *FAM129B* | 0.331 | 1.88E-06 | 2.80E-02 |  |
| *EMP3* | 0.326 | 3.64E-07 | 5.41E-03 |  |
| *S100A16* | 0.326 | 1.57E-06 | 2.33E-02 |  |
| *LMNA* | 0.323 | 2.70E-07 | 4.02E-03 |  |
| *LGALS1* | 0.303 | 9.91E-10 | 1.47E-05 |  |
| *ENO1* | 0.295 | 2.27E-10 | 3.37E-06 |  |
| *TMSB4X* | 0.290 | 2.18E-07 | 3.25E-03 |  |
| *MYL6* | 0.280 | 4.26E-09 | 6.34E-05 |  |
| *S100A6* | 0.264 | 1.27E-06 | 1.88E-02 |  |
| *HMGA1* | 0.258 | 6.66E-07 | 9.91E-03 |  |
| *MT-CO3* | -0.265 | 5.29E-08 | 7.86E-04 | **Downregulated in gH2AX^low^** |
| *EPHA2* | -0.351 | 5.63E-07 | 8.37E-03 |  |
| *FOSL1* | -0.361 | 3.83E-08 | 5.70E-04 |  |
| *CORO1C* | -0.366 | 1.08E-08 | 1.60E-04 |  |
| *MKI67* | -0.371 | 2.54E-08 | 3.78E-04 |  |
| *ANLN* | -0.382 | 1.25E-07 | 1.85E-03 |  |
| *HMGB2* | -0.385 | 1.25E-07 | 1.87E-03 |  |
| *SNX5* | -0.386 | 2.84E-07 | 4.23E-03 |  |
| *UBE2C* | -0.401 | 1.70E-07 | 2.53E-03 |  |
| *SERPINE1* | -0.491 | 2.08E-13 | 3.09E-09 |  |
| *UGCG* | -0.504 | 5.41E-09 | 8.05E-05 |  |
| *AMOTL2* | -0.513 | 2.70E-06 | 4.02E-02 |  |
| *CENPE* | -0.562 | 1.94E-09 | 2.89E-05 |  |
| *CTGF* | -0.567 | 5.09E-07 | 7.57E-03 |  |
| *CYR61* | -0.572 | 1.06E-07 | 1.57E-03 |  |
| *MDM2* | -0.643 | 1.49E-10 | 2.22E-06 |  |
| *F3* | -0.746 | 4.02E-09 | 5.98E-05 |  |
| *ANKRD1* | -0.933 | 3.75E-08 | 5.59E-04 |  |
| *KRTAP2-3* | -0.940 | 7.40E-14 | 1.10E-09 |  |

**Table S2: Differentially expressed genes in γH2AX^low^ versus γH2AX^high^ control cells**

| **Gene** | **avg_log2FC** | **p_val** | **p_val_adj** |  |
| --- | --- | --- | --- | --- |
| *ARL4C* | 0.573 | 1.61E-08 | 0.00024 | **Upregulated in gH2AX^low^** |
| *CCDC85B* | 0.542 | 4.76E-08 | 0.000708 |  |
| *ORAI1* | 0.494 | 3.83E-07 | 0.005695 |  |
| *SLC16A3* | 0.404 | 6.87E-07 | 0.010225 |  |
| *LMNA* | 0.390 | 3.30E-08 | 0.000491 |  |
| *TCEB2* | 0.366 | 5.71E-07 | 0.008496 |  |
| *CENPB* | 0.365 | 1.80E-06 | 0.026847 |  |
| *POLR2L* | 0.353 | 1.23E-08 | 0.000182 |  |
| *RPS28* | 0.310 | 1.84E-08 | 0.000273 |  |
| *RPL18A* | 0.302 | 1.86E-09 | 2.77E-05 |  |
| *RPS9* | 0.275 | 1.21E-06 | 0.017957 |  |
| *RPLP2* | 0.268 | 5.74E-09 | 8.54E-05 |  |
| *ANXA2* | -0.257 | 2.54E-07 | 0.00378 | **Downregulated in gH2AX^low^** |
| *GNAS* | -0.305 | 6.99E-10 | 1.04E-05 |  |
| *H3F3B* | -0.336 | 2.22E-07 | 0.003304 |  |
| *APLP2* | -0.357 | 2.86E-09 | 4.25E-05 |  |
| *CALM2* | -0.361 | 1.50E-10 | 2.24E-06 |  |
| *ARHGAP11A* | -0.374 | 1.90E-06 | 0.028252 |  |
| *CKAP5* | -0.385 | 4.05E-07 | 0.006024 |  |
| *PLOD2* | -0.412 | 3.30E-06 | 0.049128 |  |
| *BUB3* | -0.413 | 6.51E-10 | 9.68E-06 |  |
| *TXNRD1* | -0.418 | 2.08E-07 | 0.003096 |  |
| *TUBA1C* | -0.420 | 4.91E-11 | 7.31E-07 |  |
| *FAS* | -0.422 | 2.95E-07 | 0.004382 |  |
| *TRIM59* | -0.429 | 2.86E-07 | 0.004251 |  |
| *TPX2* | -0.435 | 2.94E-09 | 4.38E-05 |  |
| *CENPF* | -0.440 | 1.99E-07 | 0.002955 |  |
| *ARL6IP1* | -0.443 | 5.09E-10 | 7.58E-06 |  |
| *KIF11* | -0.444 | 3.53E-07 | 0.005245 |  |
| *CKS2* | -0.447 | 5.01E-07 | 0.007456 |  |
| *BUB1B* | -0.448 | 3.00E-06 | 0.044607 |  |
| *MKI67* | -0.451 | 2.33E-09 | 3.47E-05 |  |
| *MZT1* | -0.452 | 5.20E-07 | 0.00774 |  |
| *BUB1* | -0.453 | 2.54E-07 | 0.003785 |  |
| *CKAP2* | -0.454 | 3.32E-06 | 0.049411 |  |
| *PLK1* | -0.456 | 2.32E-09 | 3.45E-05 |  |
| *TNFRSF10B* | -0.463 | 1.61E-07 | 0.002397 |  |
| *KIF2C* | -0.464 | 4.29E-08 | 0.000638 |  |
| *CKAP2L* | -0.467 | 3.43E-08 | 0.00051 |  |
| *PRC1* | -0.473 | 1.19E-09 | 1.77E-05 |  |
| *RHEB* | -0.475 | 5.28E-08 | 0.000786 |  |
| *NUSAP1* | -0.475 | 4.97E-09 | 7.39E-05 |  |
| *UBALD2* | -0.483 | 1.00E-06 | 0.014874 |  |
| *LOX* | -0.486 | 2.44E-07 | 0.003632 |  |
| *NUF2* | -0.502 | 2.87E-08 | 0.000427 |  |
| *ASPM* | -0.502 | 4.81E-08 | 0.000716 |  |
| *BRD8* | -0.508 | 1.83E-07 | 0.002716 |  |
| *CEP55* | -0.511 | 3.68E-08 | 0.000548 |  |
| *AURKA* | -0.511 | 6.31E-09 | 9.39E-05 |  |
| *CDK1* | -0.518 | 2.75E-08 | 0.00041 |  |
| *CCNF* | -0.521 | 3.56E-09 | 5.30E-05 |  |
| *NCAPD2* | -0.531 | 2.22E-10 | 3.31E-06 |  |
| *KIF20A* | -0.532 | 1.75E-07 | 0.002603 |  |
| *SERPINE1* | -0.538 | 1.31E-07 | 0.001946 |  |
| *CDCA3* | -0.543 | 1.09E-10 | 1.63E-06 |  |
| *CDKN1B* | -0.559 | 1.41E-09 | 2.10E-05 |  |
| *CDKN2D* | -0.560 | 8.00E-07 | 0.011893 |  |
| *HJURP* | -0.573 | 1.74E-10 | 2.59E-06 |  |
| *TOP2A* | -0.617 | 6.32E-13 | 9.40E-09 |  |
| *UBE2C* | -0.618 | 1.76E-12 | 2.62E-08 |  |
| *ANLN* | -0.627 | 1.44E-14 | 2.14E-10 |  |
| *IGFBP7* | -0.679 | 3.23E-12 | 4.81E-08 |  |
| *HMGB2* | -0.730 | 1.45E-17 | 2.16E-13 |  |
| *CENPE* | -0.735 | 7.54E-14 | 1.12E-09 |  |

**Table S3: Enrichr results of downregulated genes in γH2AX^low^ HRAS^G12V^ cells**

| **Term** | **Overlap** | **Adjusted P-value** | **Odds Ratio** | **Combined Score** | **Genes** |
| --- | --- | --- | --- | --- | --- |
| SMAD2 18955504 ChIP-ChIP HaCaT Human | 10/1513 | 7.90E-05 | 13.66008723 | 205.5306545 | FOSL1;ANLN;SERPINE1;MDM2;ANKRD1;AMOTL2;F3;SNX5;CORO1C;EPHA2 |
| SMAD3 18955504 ChIP-ChIP HaCaT Human | 10/1513 | 7.90E-05 | 13.66008723 | 205.5306545 | FOSL1;ANLN;SERPINE1;MDM2;ANKRD1;AMOTL2;F3;SNX5;CORO1C;EPHA2 |
| FOXM1 25889361 ChIP-Seq OE33 AND U2OS Human | 7/784 | 8.33E-04 | 14.41741742 | 177.1305332 | ANLN;CENPE;UBE2C;HMGB2;MDM2;MKI67;SNX5 |
| ATF3 23680149 ChIP-Seq GBM1-GSC Human | 9/1652 | 0.001019262 | 10.04516129 | 118.4907259 | FOSL1;UBE2C;SERPINE1;HMGB2;ANKRD1;AMOTL2;F3;SNX5;EPHA2 |
| FOXM1 23109430 ChIP-Seq U2OS Human | 4/226 | 0.005827247 | 23.73453453 | 233.2912981 | UBE2C;HMGB2;MKI67;SNX5 |
| FOXA1 26769127 Chip-Seq PDAC-Cell Line Human | 8/1658 | 0.006501454 | 8.079779614 | 77.06000346 | FOSL1;UGCG;HMGB2;ANKRD1;AMOTL2;F3;SNX5;EPHA2 |
| TRP63 18441228 ChIP-ChIP KERATINOCYTES Mouse | 3/99 | 0.008317393 | 38.83789063 | 354.8585255 | FOSL1;UGCG;MDM2 |
| ATF3 27146783 Chip-Seq COLON Human | 7/1628 | 0.033175605 | 6.607032696 | 50.34504865 | FOSL1;ANLN;UBE2C;HMGB2;AMOTL2;CORO1C;EPHA2 |
| TP53 22127205 ChIP-Seq IMR90 Human | 5/766 | 0.036324836 | 9.020086353 | 66.85190362 | FOSL1;UGCG;MDM2;AMOTL2;EPHA2 |
| KLF4 18358816 ChIP-ChIP MESCs Mouse | 6/1238 | 0.040523981 | 7.023851149 | 50.54851817 | FOSL1;ANLN;UBE2C;AMOTL2;CORO1C;EPHA2 |
| **Term** | **Overlap** | **Adjusted P-value** | **Odds Ratio** | **Combined Score** | **Genes** |
| FOXM1 ENCODE | 3/95 | 0.006187925 | 40.53464674 | 375.3322941 | UBE2C;HMGB2;MKI67 |
| SALL4 CHEA | 4/355 | 0.009955127 | 14.91358025 | 120.6644002 | ANLN;F3;CYR61;CTGF |
| FOSL2 ENCODE | 3/196 | 0.014803526 | 19.22409326 | 137.0859687 | ANLN;ANKRD1;F3 |
| FOXA1 ENCODE | 3/205 | 0.014803526 | 18.3592203 | 128.5325854 | HMGB2;MDM2;ANKRD1 |
| CHD1 ENCODE | 4/655 | 0.027821368 | 7.918074757 | 46.03971427 | ANLN;CENPE;HMGB2;MDM2 |
| FOXA2 ENCODE | 3/316 | 0.027821368 | 11.78194888 | 67.91362989 | SERPINE1;MDM2;ANKRD1 |
| TP53 CHEA | 3/319 | 0.027821368 | 11.66831487 | 66.94781437 | FOSL1;MDM2;EPHA2 |
| SIN3A ENCODE | 5/1131 | 0.027821368 | 5.980398376 | 33.95010358 | FOSL1;ANLN;CENPE;HMGB2;MDM2 |
| GATA2 CHEA | 4/772 | 0.038792845 | 6.671180556 | 34.86814029 | SERPINE1;HMGB2;F3;EPHA2 |
| TP63 CHEA | 5/1399 | 0.054947757 | 4.761990162 | 22.72981048 | FOSL1;ANLN;MDM2;F3;CORO1C |

**Table S4: Enrichr results of downregulated genes in γH2AX^low^ Control cells**

| **Term** | **Overlap** | **Adjusted P-value** | **Odds Ratio** | **Combined Score** | **Genes** |
| --- | --- | --- | --- | --- | --- |
| FOXM1 25889361 ChIP-Seq OE33 AND U2OS Human | 42/784 | 6.85E-41 | 49.38422 | 4889.526 | TOP2A;CDKN1B;ARL6IP1;CDCA3;CCNF;HMGB2;HJURP;BUB1B;KIF11;MKI67;CENPB; AURKA;TUBA1C;NUF2;LMNA;NUSAP1;BUB3;BRD8;BUB1;CEP55;CDKN2D;CKAP2L; UBE2C;PLK1;CKAP2;UBALD2;CKAP5;MZT1;ASPM;ANLN;CENPE;TPX2;CENPF;PRC1; RHEB;CKS2;CDK1;KIF2C;TRIM59;NCAPD2;KIF20A;CALM2 |
| FOXM1 23109430 ChIP-Seq U2OS Human | 30/226 | 2.13E-39 | 88.86555 | 8431.663 | TOP2A;ARHGAP11A;ARL6IP1;CCNF;HMGB2;HJURP;KIF11;MKI67;AURKA;TUBA1C; NUF2;NUSAP1;CEP55;CDKN2D;CKAP2L;UBE2C;PLK1;CKAP2;CKAP5;MZT1;ASPM;TPX2;CENPF;PRC1;CDK1;KIF2C;TRIM59;NCAPD2;KIF20A;CALM2 |
| FOXM1 26456572 ChIP-Seq MCF-7 Human BreastCancer | 29/478 | 5.91E-28 | 35.96074 | 2449.843 | TOP2A;ARL6IP1;CCNF;HMGB2;HJURP;KIF11;MKI67;TUBA1C;NUF2;LMNA;CEP55; CDKN2D;CKAP2L;UBE2C;PLK1;CKAP2;UBALD2;MZT1;ASPM;TPX2;CENPF;PRC1;RHEB; CKS2;CDK1;KIF2C;TRIM59;NCAPD2;KIF20A |
| MYBL2 22936984 ChIP-ChIP MESCs Mouse | 28/1419 | 8.17E-14 | 10.36944 | 365.8033 | TOP2A;ARHGAP11A;ARL6IP1;CDCA3;HMGB2;BUB1B;KIF11;AURKA;NUF2;NUSAP1; BUB3;BRD8;RPS9;CKAP2L;UBE2C;PLK1;ANLN;CENPE;TPX2;RPS28;PRC1;RHEB;CKS2; GNAS;KIF2C;NCAPD2;KIF20A;CALM2 |
| E2F4 17652178 ChIP-ChIP JURKAT Human | 19/608 | 3.77E-12 | 13.86882 | 433.0123 | TOP2A;CDKN2D;CDCA3;PLK1;HMGB2;BUB1B;KIF11;MKI67;ASPM;ANLN;TPX2;CENPF;NUSAP1;KIF2C;KIF20A;BUB3;BRD8;BUB1;CALM2 |
| MYC 18358816 ChIP-ChIP MESCs Mouse | 29/2369 | 2.77E-09 | 6.230574 | 152.2806 | TOP2A;ARHGAP11A;ARL6IP1;CDCA3;CCNF;BUB1B;KIF11;AURKA;RPL18A;CCDC85B; NUSAP1;RPLP2;BRD8;CDKN2D;RPS9;CKAP2L;UBE2C;CKAP2;ASPM;ANLN;TPX2;PRC1; RHEB;CKS2;GNAS;KIF2C;TRIM59;NCAPD2;KIF20A |
| E2F1 18555785 ChIP-Seq MESCs Mouse | 32/3015 | 5.32E-09 | 5.683205 | 134.3161 | ARHGAP11A;ARL6IP1;HMGB2;BUB1B;CENPB;AURKA;TUBA1C;RPL18A;LMNA; NUSAP1;RPLP2;IGFBP7;SLC16A3;CEP55;RPS9;ANXA2;CKAP2L;UBE2C;APLP2;PLK1; CKAP2;TNFRSF10B;ASPM;CENPE;TPX2;RPS28;PRC1;CKS2;KIF2C;TRIM59;KIF20A; CALM2 |
| E2F4 21247883 ChIP-Seq LYMPHOBLASTOID Human | 28/2422 | 2.14E-08 | 5.699155 | 126.0016 | ARHGAP11A;CDKN1B;ARL6IP1;CDCA3;CCNF;BUB1B;KIF11;MKI67;TUBA1C;CCDC85B;NUF2;NUSAP1;RPLP2;BUB3;CEP55;POLR2L;ANXA2;APLP2;PLK1;CKAP2;CKAP5;ASPM;CENPF;PRC1;ORAI1;FAS;KIF2C;NCAPD2 |
| KDM5B 21448134 ChIP-Seq MESCs Mouse | 31/3030 | 2.62E-08 | 5.305273 | 115.5906 | TOP2A;ARHGAP11A;CCNF;HJURP;BUB1B;KIF11;MKI67;AURKA;NUSAP1;BUB3;BRD8; BUB1;CDKN2D;CKAP2L;APLP2;TXNRD1;CKAP2;CKAP5;ASPM;ANLN;CENPE;TPX2; CENPF;PRC1;RHEB;CDK1;KIF2C;TRIM59;NCAPD2;KIF20A;CALM2 |
| MYC 19079543 ChIP-ChIP MESCs Mouse | 18/1013 | 1.12E-07 | 7.44894 | 150.7122 | ARHGAP11A;RPS9;TXNRD1;CCNF;HMGB2;AURKA;ARL4C;RPS28;RPL18A;LMNA;CKS2;GNAS;RPLP2;KIF2C;TRIM59;KIF20A;SLC16A3;CALM2 |
|  |  |  |  |  |  |
| **Term** | **Overlap** | **Adjusted P-value** | **Odds Ratio** | **Combined Score** |  |
| FOXM1 ENCODE | 26/95 | 4.29E-43 | 197.0031 | 20102.84 | ARL6IP1;CCNF;HMGB2;HJURP;KIF11;MKI67;NUF2;BRD8;CEP55;CDKN2D;CKAP2L; UBE2C;PLK1;CKAP2;UBALD2;CKAP5;MZT1;ASPM;TPX2;CENPF;PRC1;RHEB;CDK1; TRIM59;KIF20A;CALM2 |
| E2F4 ENCODE | 27/710 | 4.82E-21 | 20.57026 | 1040.382 | TOP2A;ARHGAP11A;CDCA3;CCNF;HMGB2;HJURP;BUB1B;KIF11;MKI67;AURKA;NUF2;NUSAP1;BRD8;BUB1;CEP55;CKAP2L;PLK1;CKAP2;MZT1;ASPM;TPX2;CENPF;PRC1; CDK1;KIF2C;NCAPD2;KIF20A |
| NFYB ENCODE | 42/3715 | 4.99E-15 | 8.452912 | 307.0211 | TOP2A;CDKN1B;ARL6IP1;CDCA3;CCNF;HMGB2;HJURP;KIF11;MKI67;AURKA;TUBA1C;RPL18A;LMNA;NUSAP1;RPLP2;BUB3;BRD8;BUB1;CEP55;CDKN2D;RPS9;H3F3B; CKAP2L;UBE2C;PLK1;CKAP2;TNFRSF10B;UBALD2;CKAP5;MZT1;ASPM;CENPE;TPX2; CENPF;PRC1;RHEB;CKS2;CDK1;TRIM59;NCAPD2;KIF20A;CALM2 |
| SIN3A ENCODE | 25/1131 | 5.32E-14 | 10.91366 | 367.4376 | HMGB2;HJURP;BUB1B;KIF11;AURKA;TUBA1C;CCDC85B;NUF2;BRD8;BUB1;CEP55; POLR2L;CKAP2L;PLK1;UBALD2;ANLN;CENPE;TPX2;CENPF;CKS2;CDK1;KIF2C;NCAPD2;KIF20A;CALM2 |
| NFYA ENCODE | 30/2250 | 1.74E-11 | 7.041335 | 194.7045 | TOP2A;ARL6IP1;CDCA3;CCNF;HMGB2;HJURP;AURKA;RPL18A;NUSAP1;RPLP2;BUB3; BRD8;BUB1;CDKN2D;H3F3B;CKAP2L;CKAP2;UBALD2;CKAP5;MZT1;ASPM;TPX2; CENPF;PRC1;RHEB;CKS2;CDK1;NCAPD2;KIF20A;CALM2 |
| IRF3 ENCODE | 13/663 | 2.13E-06 | 7.563137 | 119.1525 | TOP2A;CDCA3;H3F3B;CKAP2L;UBE2C;HMGB2;UBALD2;MZT1;AURKA;TPX2;RPL18A; BUB3;BUB1 |
| NELFE ENCODE | 8/234 | 9.97E-06 | 12.45891 | 175.1524 | TUBA1C;RPS9;RPS28;ARL6IP1;H3F3B;HMGB2;NUSAP1;CALM2 |
| ZMIZ1 ENCODE | 13/914 | 5.83E-05 | 5.385193 | 65.47722 | CDKN1B;ARL6IP1;H3F3B;APLP2;HMGB2;KIF11;MZT1;TUBA1C;RPS28;CCDC85B;LMNA;NCAPD2;CALM2 |
| SP1 ENCODE | 11/707 | 1.31E-04 | 5.737367 | 64.36025 | TOP2A;ASPM;ARL6IP1;CDCA3;H3F3B;RHEB;HMGB2;HJURP;UBALD2;CKAP5;BUB1 |
| E2F1 CHEA | 12/859 | 1.31E-04 | 5.20089 | 57.67517 | ARHGAP11A;CDKN1B;ARL6IP1;RPL18A;H3F3B;UBE2C;HMGB2;BUB1B;RPLP2;CALM2;CENPB;AURKA |

**Table S5. Key resources**

| **Reagent or resource** | **Source** | **Identifier** |
| --- | --- | --- |
| **Antibodies** | | |
| CHK1 phospho S296 | Cell Signaling Technology | 2349;  RRID:AB_2080323 |
| CHK1 phospho S345 | Cell Signaling Technology | 2348;  RRID:AB_331212 |
| CHK1 | Cell Signaling Technology | 2360; RRID:AB_2080320 |
| γ-H2AX (S139) | Cell Signaling Technology | 2577;  RRID:AB_2118010 |
| γ-TUBULIN | Sigma-Aldrich | T6557; RRID:AB_477584 |
| CHK1 phospho S345 - PE conjugated | Abcam | Ab278744;  RRID:AB_2745001 |
| KAP1 phospho S824 | Bethyl | A300-767A;  RRID: AB_669740 |
| RPA2 phospho S8 | Cell Signaling Technology | 54762S;  RRID: AB_2799471 |
| γH2AX – Alexa647 conjugated | BioLegend | 613407;  RRID: AB_2114994 |
| pFOXM1 T600 | Cell Signaling Technology | 14655;RRID:AB_2798557 |
| **Chemicals** | | |
| Palbociclib | Selleck chemicals | S1116 |
| Gemcitabine | Selleck chemicals | S1714 |
| Prexasertib | Selleck chemicals | S7178 |
| DAPI | Sigma-Aldrich | D9542 |
| Protease inhibitor Cocktail | Sigma-Aldrich | 11873580001 |
| Fetal bovine serum | Thermo Fisher | 10500064 |
| DMEM | Thermo Fisher | 41966052 |
| **Critical commercial assays** | | |
| Rneasy Micro Kit for RNA extraction | Qiagen | 74004 |
| **Deposited data** | | |
| Single-cell RNA sequencing RPE-FUCCI4 HRAS^G12V^ cells | This paper |  |
| **Experimental Models** | | |
| hTERT RPE-1 cells | ATCC | CRL-4000;  RRID:CVCL_4388 |
| **Oligonucleotides** | | |
| Primers used for qPCR, see Table S3 | This paper | Biolegio |
| Scrambled siRNA | Dharmacon | D-001210-02-05 |
| **Software and Algorithms** | | |
| FIJI (ImageJ) | <https://fiji.sc> | RRID:SCR_002285 |
| FlowJo | BD | RRID:SCR_008520 |
| R | <https://www.R-project.org/> | RRID:SCR_001905 |
| Rstudio | <https://www.rstudio.com/> | RRID:SCR_000432 |
| Seurat | https://github.com/satijalab/seurat | RRID:SCR_016341 |

**Table S6. Antibodies for immunoblots and immunofluorescence staining**

| ***Application*** | ***Name*** | ***Company*** | ***Catalogue number*** | ***Dilution*** |
| --- | --- | --- | --- | --- |
| *Immunoblots* | CHK1 phospho S296 | Cell Signaling | 2349 | 1:1000 |
|  | CHK1 phospho S345 | Cell Signaling | 2348 | 1:1000 |
|  | CHK1 | Cell Signaling | 2360 | 1:1000 |
|  | KAP1 phospho S824 | Bethyl | A300-767A | 1:500 |
|  | γ-H2AX (S139) | Cell Signaling | 2577 | 1:1000 |
|  | γ-TUBULIN | Sigma-Aldrich | T6557 (GTU-88) | 1:1000 |
| *Immunofluorescence* | γ-H2AX (S139) | Cell Signaling | 2577 | 1:200 |
| *Flow cytometry* | CHK1 phospho S345 – PE conjugated | Abcam | Ab278744 | 1:5000 |
|  | KAP1 phospho S824 | Bethyl | A300-767A | 1:500 |
|  | RPA2 phospho S8 | Cell Signaling | 54762S | 1:500 |
|  | γH2AX – Alexa647 conjugated | BioLegend | 613407 | 1:200 |
|  | pFOXM1 T600 | Cell Signaling | 14655 | 1:500 |

**Table S7. qPCR primers**

| **Gene** | **Forward primer (5’-3’)** | **Reverse primer (3’-5’)** |
| --- | --- | --- |
| *AMOTL2* | CTACAGCAGACAGAGCACCC | ATCTCTGCTCCCGTGTTTGG |
| *ANKRD1* | CGGAGCATCTTATCGCCTGT | TTCTGCCAGTGTAGCACCAG |
| *ARL4C* | TTGGTTCGCTCTTTGTTCGC | GAAACGCAGGAAGTCCCTCA |
| *β-ACTIN* | GATCGGCGGCTCCATCCTG | GACTCGTCATACTCCTGCTTGC |
| *COPE* | GCCATGACAGTGCAGATCCT | GCACTTGTCAGCCATCTCCT |
| *CORO1* | TTTGCAGCGGGGACTTCG | TTGCTCTGTCGTACCACTCG |
| *CTGF* | ATGGTGCTCCCTGCATCTTC | CTGGTACTTGCAGCTGCTCT |
| *CYR61* | CCAGTGTACAGCAGCCTGAA | CGCATCTTCACAGTCCTGGT |
| *ENO1* | GGCTGTTGAGCACATCAATAAAAC | GCACCAGCTTTGCAGACG |
| *EMP3* | CGAGGGACAAGACTCCGAC | TTGTCCAAAGTGGCCACGAA |
| *EPHA2* | TCACACACCCGTATGGCAAA | ACGTTGCACACGGAGTACAT |
| *F3* | CAGCCCGGTAGAGTGTATGG | AGCTCCAACAGTGCTTCCTT |
| *FAM129B* | CCCTTCCTTTTGGGGCTCTC | AAGAGAGCCACGCCATACTG |
| *FOXM1* | AGACCTGTGCAGATGGTGAG | CTGATGGTCTCGAAGGCTCC |
| *FSCN1* | AAGGACGAGCTCTTTGCTCT | CGGTCTCCTCGTCCTGATTG |
| *GAPDH* | CTCTGCTCCTCCTGTTCG | GCCCAATACGACCAAATCC |
| *GNB2* | ACAGTGGGTTTTGCTGGACA | CGTAGCCGTTGGGGAAGAAA |
| *IER2* | GTGTCGGAGTTCTGTCTGGG | CCAAACACTCATTGCCCGTG |
| *LGALS1* | TCGGGTGGAGTCTTCTGACA | CAGGTTCAGCACGAAGCTCT |
| *LMNA* | GCGTACGGCTCTCATCAACT | CGAGCGCAGGTTGTACTCA |
| *HMGA1* | GCTCCTCTAATTGGGACTCCG | TCCTTTTCCTGCTTGGAGGC |
| *MAP4K4* | TTTTCAGACCCCTCAAGCCT | TTGTGAGGTGGCCGTACATC |
| *MYADM* | ACAGCCTGTTCCAAGTGTGG | AAGATGACGTCGTGGTGGTT |
| *MYL6* | GTCGAAGGACTTCGGGTGTT | AAGGTCCTCAGCCATTCAGC |
| *KRTAP2-3* | AGCTGATCCTCAAGCACGAA | GGGTGATGAGTCAGTGGGAC |
| *PRDX5* | ACGCTCAGCGGGCTATATACT | TCAAACACCTCCACTGCTGG |
| *RSP18* | AGTTCCAGCATATTTTGCGAG | CTCTTGGTGAGGTCAATGTC |
| *S100A16* | CCAAATCTGACTGTGGCTTGC | CTGACATCTCCCTGCTTCGC |
| *S100A6* | ATTTGGCCGCCTCCCTACC | TTCTTGCTCAGGGTGTGCTT |
| *TMSB4X* | CCAGACTTCGCTCGTACTCG | CGCACGCCTCATTACGATTC |
| *UGCG* | TTGCTGCCACCTTAGAGCAG | TCGGTCAGCTATCGCTTTGG |

1. **Supplementary Figure Legends**

**Figure S1. Related to Figure 2**

**A** Violin plot showing the number of unique RNA (UMI) counts per cell in DSP-fixed and fresh cells RPE-HRAS^G12V^ cells.

**B** Violin plot showing the percentage of RNA counts mapping to mitochondrial genes as percentage of total counts detected in DSP-fixed and fresh cells RPE-HRAS^G12V^ cells.

**C** Violin plot showing ERCC spike-in RNA counts as percentage of total counts detected in DSP-fixed and fresh RPE-HRAS^G12V^ cells.

**Figure S2. Related to Figure 4**

**A** The DAPI fluorescence intensity in individual FACS-sorted cells shown in a violin plot. Horizontal lines represent median DAPI levels in each group. Wilcoxon rank sum tests for multiple group comparisons were performed on control cells and HRAS^G12V^ cells separately. *** P<2E-16 γH2AX-negative versus all other groups, * P<0.005 γH2AX^low^ versus γH2AX^medium^ and γH2AX^high^.

**B** Correlation matrix displaying the correlations in normalized transcript counts between all the differentially expressed genes in RPE HRAS^G12V^ γH2AX^high^ versus γH2AX^low^ cells. Highlighted genes show a correlation coefficient greater than 0.4 with at least 1 other gene.

**C** Heatmap of genes differentially expressed and downregulated in γH2AX^low^ versus γH2AX^high^ control RPE cells after treatment with 10 nM CHK1i + 4 nM gemcitabine. The heatmaps represent normalized transcript counts in single cell RNA-sequencing analysis.

**D** Heatmap of genes differentially expressed and upregulated in γH2AX^low^ versus γH2AX^high^ control RPE cells after treatment with 10 nM CHK1i + 4 nM gemcitabine. The heatmaps represent normalized transcript counts in single cell RNA-sequencing analysis.
Plots in **C** and **D** represent n=141, 194, and 214 cells in the γH2AX^low^, γH2AX^medium^, and γH2AX^high^ groups, respectively.

**Figure S3. Related to Figure 5A**

**A** Quantitative PCR of the expression of potential RS-tolerance conferring genes in RPE-HRAS^G12V^ cells treated with scrambled siRNA or siRNA targeting the gene of interest. Gene expression was normalized to the average of two housekeeping genes (*GAPDH, 18S).* Bar represents mean ± s.e.m.

**B** Flow cytometry data of RPE-HRAS^G12V^ cells unsynchronized, arrested in G1-phase after 24 hours treatment with a CDK4/6i and at indicated hours after release in the presence and absence of CHK1i + gemcitabine to enrich for S/G2-phase cells. DAPI staining was used to determine cell cycle progression (top row). The relationship between Geminin_1-110_ and DAPI is shown in the bottom row. Representative of 2 independent experiments.

**C** Flow cytometry data of RPE-HRAS^G12V^ cells with the indicated genes depleted by Smartpools of four individual siRNAs. DAPI staining was used to determine cell cycle progression (top row) and γH2AX staining was used to determine the degree of replication stress (bottom row). Number in bottom right corner of bottom row plots indicates the Geminin_1-110_^+^ cells as percentage of the total cells. Number in the top right corner of bottom row plots indicates γH2AX^+^ cells as a percentage of Geminin_1-110_^+^ cells. Representative of 2 independent experiments.

**Figure S4. Related to Figure 5C and 5E**

**A** Quantification of percent of γH2AX-positive cells in geminin-positive cells (representative of cells in S/G2 phase) from two individual experiments. Error bars indicate mean +/- SEM. *p<0.01.

**B** Raw IntensityPercent values calculated using the ImageJ ColonyArea plug-in. Error bars indicate mean +/- SEM. Significant differences were determined by ordinary One-way ANOVA followed by Dunnett’s multiple comparison test. *p<0.05, **p<0.01, N=3.

**Figure S5. Related to Figure 5.**

**A** DNA damage, measured by γH2AX flow cytometry in S and G2 RPE-HRAS^G12V^ cells treated with indicated FOXM1 target genes depleted by Smartpools of four individual siRNAs. DAPI staining was used to determine cell cycle progression (top row) and γH2AX staining was used to determine the degree of replication stress (bottom row). Number in bottom right corner of bottom row plots indicates the Geminin_1-110_^+^ cells as percentage of the total cells. Number in the top right corner of bottom row plots indicates γH2AX^+^ cells as a percentage of Geminin_1-110_^+^ cells.

**B** Quantitative PCR of the expression of FOXM1 target genes in RPE-HRAS^G12V^ cells treated with scrambled siRNA or siRNA Smartpools targeting the gene of interest. Gene expression was normalized to the average of 2 reference genes (*GAPDH, 18S*). Bars represent mean ± s.e.m.

**C** DNA damage, measured by γH2AX flow cytometry in G2 RPE-HRAS^G12V^ cells treated with four individual siRNAs targeting the FOXM1 target genes *MKI67* and *UBE2C*. DAPI staining was used to determine cell cycle progression (top row) and γH2AX staining was used to determine the degree of replication stress (bottom row). Number in bottom right corner of bottom row plots indicates the Geminin_1-110_^+^ cells as percentage of the total cells. Number in the top right corner of bottom row plots indicates γH2AX^+^ cells as a percentage of Geminin_1-110_^+^ cells.

**D** Quantitative PCR of the expression of the FOXM1 target genes *MKI67* and *UBE2C* in RPE-HRAS^G12V^ cells treated with scrambled siRNA or 4 different individual siRNAs targeting the gene of interest. Gene expression was normalized to the average of 2 reference genes (*GAPDH, 18S*). Bars represent mean ± s.e.m.
